# Effect of Polymer Molecular Weight on the Structure and Properties of Ultra-High-Molecular-Weight Polyethylene Membranes Prepared via Controlled Swelling

**DOI:** 10.3390/polym17152044

**Published:** 2025-07-26

**Authors:** Andrey V. Basko, Konstantin V. Pochivalov, Tatyana N. Lebedeva, Mikhail Y. Yurov, Alexander S. Zabolotnov, Sergey S. Gostev, Alexey A. Yushkin, Alexey V. Volkov, Sergei V. Bronnikov

**Affiliations:** 1 G A Krestov Institute of Solution Chemistry, Russian Academy of Sciences, ul. Akademicheskaya, 1, Ivanovo 153045, Russia; avb@isc-ras.ru (A.V.B.);; 2 N N Semenov Institute of Chemical Physics, Russian Academy of Sciences, ul. Kosygina, 4, Moscow 119991, Russia; 3 A V Topchiev Institute of Petrochemical Synthesis, Russian Academy of Sciences, Leninsky pr., 29-2, Moscow 119071, Russia; 4 Branch of Petersburg Nuclear Physics Institute named by B.P. Konstantinov of National Research Centre “Kurchatov Institute” Institute of Macromolecular Compounds, Bolshoy pr. of V.O., 31, Saint Petersburg 199004, Russia

**Keywords:** ultra-high-molecular-weight polyethylene, swelling, ultrafiltration membrane, thermally induced phase separation

## Abstract

A recently proposed method called “controlled swelling of monolithic films” was implemented to prepare ultra-high-molecular-weight polyethylene (UHMWPE) ultrafiltration membranes. For the first time, the effect of UHMWPE molecular weight (MW) on the structure and properties of the membranes prepared via this special case of thermally induced phase separation was studied in detail. The morphology and properties of the membranes were studied using SEM, DSC, liquid–liquid displacement porometry, and standard methods for the evaluation of mechanical properties, permeance, rejection, and abrasion resistance. High-quality membranes with a tensile strength of 5.0–17.8 MPa, a mean pore size of 25–50 nm, permeance of 17–107 L m^−2^ h^−1^ bar^−1^, rejection of model contaminant (blue dextran) of 72–98%, and great abrasion resistance can be prepared only if the MW of the polymer in the initial monolithic film is sufficiently high. The properties of the membranes can effectively be controlled by changing the MW of the polymer and the mass fraction of the latter in the swollen film. Shrinkage is responsible for the variation in the membrane properties. The membranes prepared from a higher-MW polymer are more prone to shrinking after the removal of the solvent. Shrinkage decreases before rising again and minimizes with an increase in the polymer content in the swollen film.

## 1. Introduction

Membrane technologies have been applied in different areas, varying from gas separation [1], liquid filtration [2], and water treatment [3,4] to battery separators [5], membrane crystallization devices [6], and the preparation of microparticles [7]. Among the polymers used for membrane preparation, polyvinylidene fluoride [8], polysulphone [9], cellulose acetate [10], polyetherketone [11], and polyolefins, particularly polyethylene [12], polypropylene [13], and poly-4-methyl-1-pentene [14], are the most widespread.

In recent years, an interest in preparing membranes using ultra-high-molecular-weight polyethylene (UHMWPE) has been growing [15]. This is due to its high tensile and puncture strength [16], chemical resistance, and wear resistance [17].

One of the first papers in which attention was paid to the abrasion resistance of membranes used for the filtration of suspensions was published in 1999 [18]. It was shown that magnesium and calcium phosphate crystals can cause abrasion of ceramic membranes in bioreactors, thus leading to a decrease in their permeance. The problem of an increase in permeance, along with a decrease in selectivity and mechanical properties in the polymeric membranes due to their abrasion, has been intensively studied [19,20]. Later, several dozen papers, in which polymeric membranes were prepared and characterized in terms of wear resistance, were published [21,22,23,24,25,26,27,28,29,30,31,32,33].

In [22,23], it was shown that the abrasion resistance of the membranes, assessed via the mass loss after treatment with sandpaper or storage in a continuously mixed suspension of silicon carbide, may be improved by adding a small amount of nanoclay to the dope solution. A similar method was also applied in [24], but the amount of nanoclay added was much higher (up to 10%). In ref. [25], it was shown that prefiltration of molasses distillery wastewater through filter paper before the main filtration process using a polymeric membrane negates the abrasion effect of the feed mixture components on membrane stability. In order to prepare abrasion-resistant polyvinylidene fluoride membranes for membrane distillation, adding microsilica to the dope solution for electrospinning was proposed [26]. The addition of ~3 wt.% microsilica enabled the production of superhydrophobic membranes that maintained their properties after 160 h of continuous filtration. A positive effect of nanoparticle (graphene oxide and nanodiamonds) addition on the abrasion resistance of polyvinyl chloride membranes was also observed in [27].

It should be noted that nanoparticle addition does not always improve the abrasion resistance of a membrane. For example, in ref. [28], the authors showed that the addition of titanium dioxide to the dope solution increases the permeance of a membrane but decreases its abrasion resistance. In ref. [29], composite membranes based on polyvinyl chloride and polycarbonate were prepared, and it was shown that an increase in the content of the latter in the membranes not only improved their strength and abrasion resistance but also increased their permeance. An increase in polyurethane additive in composite membranes based on polyvinyl chloride was shown to increase their abrasion resistance [30]. It was also proposed to protect the active, easily abraded material (polyacrylamide membrane) by sandwiching it between the cotton fabric materials [31]. Zirconium-based metal–organic frameworks were added to the collagen fiber membrane to form a highly effective and abrasion-resistant membrane from genuine leather [32]. Superhydrophobic membranes were prepared by forming a layer of spherulitic structures on the surface of the membranes, and it was shown that their hydrophobicity was maintained even after abrasion resistance tests and ultrasound treatment [33]. More details on the abrasion resistance and overall durability of the membranes for liquid filtration were discussed in a review paper [34]. At the same time, despite UHMWPE having an advantage in abrasion resistance, as shown in a literature analysis, to date, there are no papers in which the abrasion resistance of UHMWPE membranes is reported.

A few methods can be used for UHMWPE membrane preparation, including stretching, powder sintering, and thermally induced phase separation (TIPS). The TIPS method is the most widely developed method for UHMWPE membrane formation. But this method is complicated since it is difficult to form a homogeneous dope solution with the required concentration [15]. That is why, in recent years, several original methods for preparing UHMWPE membranes have been developed [35,36,37].

In our recent paper, we also proposed a new method of UHMWPE membrane preparation based on the controlled swelling of monolithic films and discussed the peculiarities of the structure formation mechanism [38]. This method consists of monolithic film annealing at an elevated temperature in a large amount of solvent (xylene), followed by cooling the swollen film to room temperature, extracting the solvent, and, finally, drying. Membranes’ morphologies and properties can effectively be controlled by changing the parameters of the membrane formation process, such as the cooling rate, thermodynamic affinity of dope solution components, and polymer MW [15]. In works published in the 2000s [39,40,41], it was shown that increasing polymer MW decreases the pore size in polypropylene membranes prepared via TIPS. Later, this tendency was also observed for polyvinylidene fluoride membranes [42]. In ref. [43], it was shown that increasing MW not only decreases the pore size but also decreases the depth of non-solvent diffusion into the dope solution in the process of non-solvent/thermally induced phase separation and corresponding changes in the formed membrane structure.

The effect of polymer MW on the structure and properties of nonporous UHMWPE materials [44,45] and peculiarities of polymer behavior [46,47] were reported. Meanwhile, the effect of polymer MW on the structure and properties of UHMWPE membranes is scarce in the literature. Particularly, it was shown [48] that an increase in UHMWPE MW from 2.5 to 4·10^6^ g mol^−1^ leads to a decrease in permeance of membranes prepared via TIPS and an increase in rejection due to the pore size decrease [48]. In ref. [49], we found that an increase in polymer MW results in a change in the phase separation type in the TIPS process from solid–liquid to liquid–liquid.

The goal of the present work is to further develop the understanding of the controlled swelling method for the preparation of UHMWPE membranes from monolithic films, to assess the prospects of using decalin as a swelling agent, and to investigate the effect of polymer MW on the morphology, transport, physico-mechanical, and tribological properties of UHMWPE membranes prepared via controlled swelling.

## 2. Materials and Methods

### 2.1. Materials

In this study, 99% decalin (Macklin, Shanghai, China), with a boiling temperature of 186 °C and a density of 0.896 g cm^−3^ at 25 °C, was used as a swelling agent, and 99.6% iso-propanol (Ekos-1, Moscow, Russia), with a boiling temperature of 82.5 °C and a density of 0.785 g cm^−3^ at 25 °C, was used as an extractant.

UHMWPE samples with different MWs were synthesized according to the technique described previously [49]. The samples were prepared via polymerization in situ using a catalyst deposited on graphite nanoplates acting as a filler in the synthesized composite UHMWPE powder. The graphite nanoplate concentration in the synthesized polymer was 0.11 wt.%. Controlling the synthesis conditions (temperature and hydrogen gas addition), three samples with different MWs were prepared. The principal characteristics of the UHMWPE samples are listed in Table 1.

The MW of the samples was detected via gel-permeation chromatography (HLC-8321 GPC/HT Tosoh, Tokyo, Japan) according to the technique described previously [49]. The melting temperature and crystallinity degree (α) of the samples were assessed using a differential scanning calorimetry (DSC) technique.

### 2.2. Membrane Preparation

The first stage of the membrane preparation was the hot pressing of the synthesized powders into a thin monolithic film. The required quantity of the powder was evenly deposited within a 150 μm thick frame on the surface of a polyethylene terephthalate film lying on a polished stainless steel plate, covered with a second polyethylene terephthalate film and a second stainless steel plate. The package was then placed between the plates of a hydraulic press heated to 170 °C. Then, a pressure of ~10 bar was applied for 10 min to ensure uniform heating of the sample. After that, the pressure was increased to ~160 bar and left for 10 min again. Finally, the sample was cooled down in the press by changing the steam circulating in the instrument plates with cold water. The mean cooling rate in the range 90–120 °C was ca. 30 °C min^−1^. As a result, monolithic films of 25 × 25 cm were obtained.

The prepared films were cut into squares and placed into a hot weighing bottle with an excess of decalin (bath modulus 1:200) for swelling. The swelling temperatures were chosen based on preliminary experiments so that the required swelling degree could be attained in a reasonable time. At lower temperatures, the swelling was too slow, whereas at higher temperatures, it tended to be nonuniform. The swelling time was adjusted in order to obtain samples with the required polymer mass fraction in swollen film (PMFSF). The latter was determined by taking into account the variation in the sample size due to its swelling. It was calculated as a ratio of the polymer mass in the swollen film to the mass of the swollen film according to Equation (1):

(1) $$PMFSF=\;m_{p}/m_{sf}=\;\left(m_{f}-m_{e}\right)/\left(\left(m_{f}-m_{e}\right)+\left(\left(a_{sf}b_{sf}d_{sf}-a_{0}b_{0}d_{0}\right)\rho_{dec}\right)\right),$$

where *m_p_* is the polymer mass in the swollen film; *m_sf_* is the mass of the swollen film; *m_f_* is the mass of the initial monolithic film; *m_e_* is the mass of the polymer extracted in the swelling process; *a_sf_*, *b_sf_*, and *d_sf_* are the dimensions of the swollen film; *a*_0_, *b*_0_, and *d*_0_ are the dimensions of the initial film; and *ρ_dec_* is the decalin density. The value of *d_sf_* was not measured but taken as increased by the same factor as *a_sf_* and *b_sf_* compared to *a*_0_ and *b*_0_.

After the required PMFSF was reached, the bottle with the sample was cooled, and thus, the swelling process was stopped. The membrane samples were transferred to iso-propanol to extract decalin for 12 h. After that, the samples were then transferred into a fresh iso-propanol bath to remove traces of decalin for 24 h and then air-dried to a constant mass.

As many as 12 samples were chosen for the following studies, with the main preparation conditions listed in Table 2.

### 2.3. Membrane and Film Characterization

In order to determine the melting temperatures and α of UHMWPE in monolithic films and membranes, DSC thermograms were recorded at a scanning rate of 10 °C min^−1^ using a 204F1 Phoenix instrument (NETZSCH, Selb, Germany). The values of *α* were calculated according to Equation (2):

(2) $$\alpha =\;{∆H}_{m}/{∆H}_{m}^{100\%},$$

where Δ*H_m_* is the melting enthalpy of the sample determined in the DSC experiment and Δ*H_m_*^100%^ = 293 J g^−1^ is the melting enthalpy of a hypothetical polyethylene sample, with *α* = 100% [50]. The melting temperature was taken as the maximum temperature of the endothermic peak in the DSC thermogram.

A DSC study of the UHMWPE mixtures with decalin was performed according to the following procedure. The required amounts of UHMWPE and decalin were placed into an aluminum pan, sealed and heated in a calorimeter from room temperature to 180 °C, annealed at this temperature for 30 min for homogenization, cooled to room temperature, thermostated for 3 min, and heated again to 180 °C. The tensile strength (*σ*) and relative elongation at break (*ε*) of the films and membranes were determined using an I-11M instrument (Tochpribor-KB, Ivanovo, Russia). The 25 × 3 mm samples with a working length of 15 mm were studied at an extension rate of 50 mm min^−1^. The mean value of *σ* was calculated from five individual measurements according to Equation (3):

(3) $$\sigma =\;F_{max}/S_{0},\;\mathrm{MPa}$$

where *F_max_* is the breaking force and *S*_0_ is the cross-section area of the sample including (for the membranes) the area of pores. The mean value of *ε* was calculated from the same five measurements according to Equation (4):

(4) $$\varepsilon =(l_{br}\;-\;l_{0})/l_{0}$$

where *l_br_* is the final length of the sample at break and *l*_0_ is the initial length of the sample.

The morphology of the surfaces and cross-section surfaces of the samples was investigated using a Quattro S scanning electron microscope (SEM) (Thermo Fischer Scientific, Brno, Czech Republic). The images were recorded from secondary electrons using a lower detector at the accelerating voltage of 1 kV. In order to investigate the cross-section morphology of the samples, they were cleaved in liquid nitrogen. The samples were coated with gold using a Quorum Q150es plus (Quorum Technologies, Lewes, UK) sputter coater prior to this study.

The porosity of the membranes was calculated using Equation (5):

(5) $$P=\left(V_{m}-V_{f}\right)/V_{m}$$

where *V_m_* and *V_f_* are the volumes of the membrane and initial film, respectively.

Filtration experiments were carried out in a dead-end stirred filtration cell. For the filtration experiments, the membrane coupon was placed onto porous stainless steel disks and sealed with a rubber O-ring. The active membrane area in the cell was 7.9 cm^2^. The system was pressurized with helium. The transmembrane pressure was 5 bar. Water was used as a solvent. Blue dextran (M_w_ = 500 kg mol^−1^) was used as a solute at a concentration of 10 mg L^−1^. Due to the hydrophobic nature of UHMWPE, ethanol was filtered through every membrane coupon for 1 h at 5 bar to fill all the membrane pores. Pure water was filtered for 2 h at 5 bar to determine the pure water permeance. Then, the blue dextran solution in water was filtered until 100 mL of permeate was collected. In the case of solution filtration, the feed was stirred at 550 rpm to minimize the concentration polarization effect. The membrane permeance *L* (L m^−2^ h^−1^ bar^−1^) was determined as:

(6) L=mρ·S·t·∆p

where ∆*p* is the transmembrane pressure (bar), *m* is the mass of the permeate (g), *ρ* is the density of the permeate (g L^−1^), *S* is the active membrane area (m^2^), and *t* is the filtration time (h).

The concentration of blue dextran in the feed and permeate was measured with a spectrophotometer at a wavelength of 620 nm. The rejection *R* was calculated using the following relation:

(7) R=1−CpCf·100%

where *C_f_* and *Cp* denote the solute concentrations in the feed and permeate, respectively.

The mean flow pore size (MFP) was measured by liquid–liquid displacement porosimetry using a POROLIQ 1000 ML porometer (Porometer, Nazareth, Belgium) according to the procedure described in ref. [51]. MFP is such a pore size that half the flux goes through pores of a higher diameter and half the flux goes through pores of a lower diameter. The measurements were carried out at room temperature using a pair of liquids prepared by demixing a mixture of isobutanol and water (1/4, *v*/*v*). The coupons (2.5 cm in diameter) were cut from the membrane and then placed into the beaker with a wetting liquid (alcohol-rich phase) for 12 h at room temperature before testing.

The abrasion resistance of the films and membranes was assessed according to the modified technique described in refs. [26,52]. Briefly, the membrane sample of a given mass was attached to a glass by double-sided tape, placed on sandpaper, and moved back and forth with a weight on top of the glass. We used the sandpaper P400 (grain size of 28–40 μm) (Zolder, Yanino-1, Russia). The loading weight was 1 kg, and the number of cycles was 200 since, at a standard weight of 200 g and 50 cycles of movement, there was almost no mass loss due to abrasion.

## 3. Results and Discussion

### 3.1. Thermal Behavior of the Decalin Mixtures of UHMWPE of Different MWs

**Figure 1:**
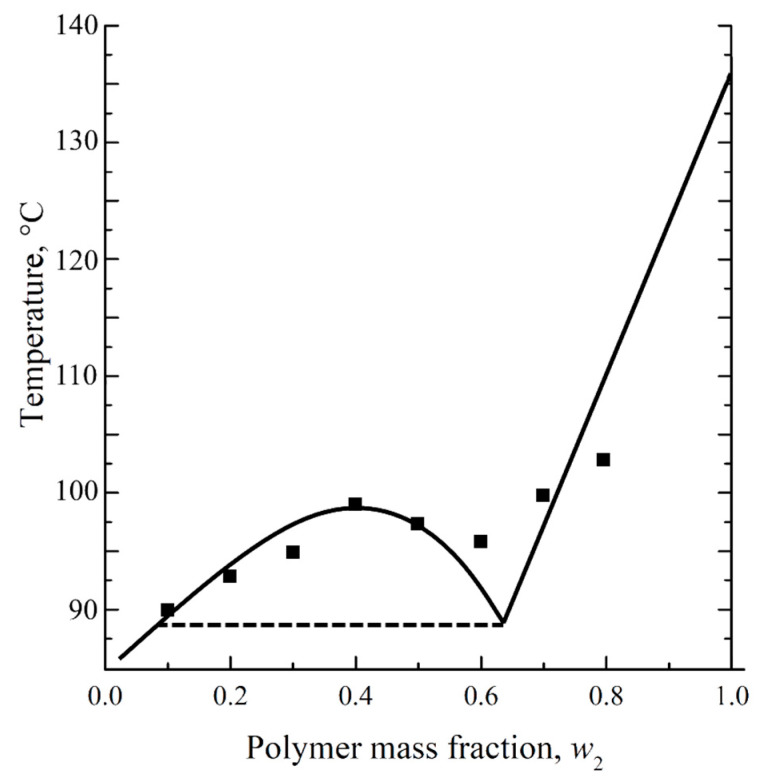
Phase diagram for the UHMWPE–decalin mixture. Adapted with permission from [56].

Decalin is known to be a good solvent for UHMWPE [53,54,55]. However, in ref. [56], a phase diagram was obtained for a mixture of UHMWPE with decalin, containing a binodal with an upper critical solution temperature (Figure 1). The points obtained experimentally were not quite in agreement with the solid lines shown on the same diagram. Furthermore, the porous sample prepared by cooling the mixture containing 40% wt. of UHMWPE and 60% wt. of decalin was shown to have a spherulitic morphology [56]. Meanwhile, the spherulitic morphology is a result of solid–liquid phase separation (i.e., polymer crystallization directly from the homogeneous mixture) rather than the liquid–liquid phase separation associated with binodal on the phase diagram.

**Figure 2:**
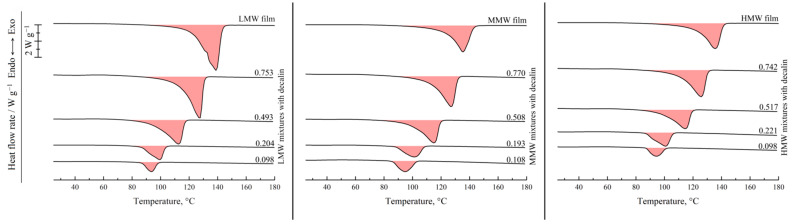
Second heating DSC thermograms of the mixtures of UHMWPE of different MW (LMW—**left**; MMW—**middle**, HMW—**right**) with decalin, together with the first heating DSC thermograms of the corresponding hot-pressed films. The polymer mass fraction in the mixtures is shown above the curves.

Figure 2 shows the results of the DSC study of the UHMWPE films and binary mixtures of UHMWPE of different MWs and decalin proportions. For the hot-pressed films, the thermograms recorded in the first heating are shown, while for the mixtures, the second heating endotherms are presented. One can see that all the DSC curves contain a single endothermic peak corresponding to the thermal effect of the UHMWPE amorphization (melting) process. The peak maximum temperature increases with an increase in the polymer content in the mixture. The area of the peak (melting enthalpy related to the overall mass of the mixture) in the mixtures containing LMW increases monotonically and passes through a maximum for the mixtures containing MMW and HMW with increasing polymer concentration in the mixture.

**Figure 3:**
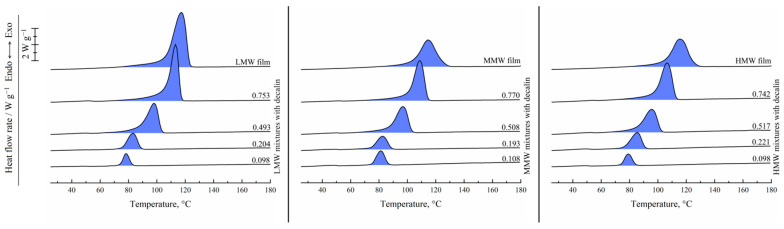
First cooling DSC thermograms of the UHMWPE of different MW (LMW—**left**; MMW—**middle**; HMW—**right**) and their mixtures with decalin. The polymer mass fraction in the mixtures is shown above the curves.

Figure 3 shows the DSC thermograms of the same films and mixtures recorded in the first cooling from 180 °C. They contain a single exothermic peak corresponding to the polymer crystallization. Its onset temperature also increases with increasing polymer concentration, and the trend of its area variation is in agreement with that observed on the second heating thermograms.

**Figure 4:**
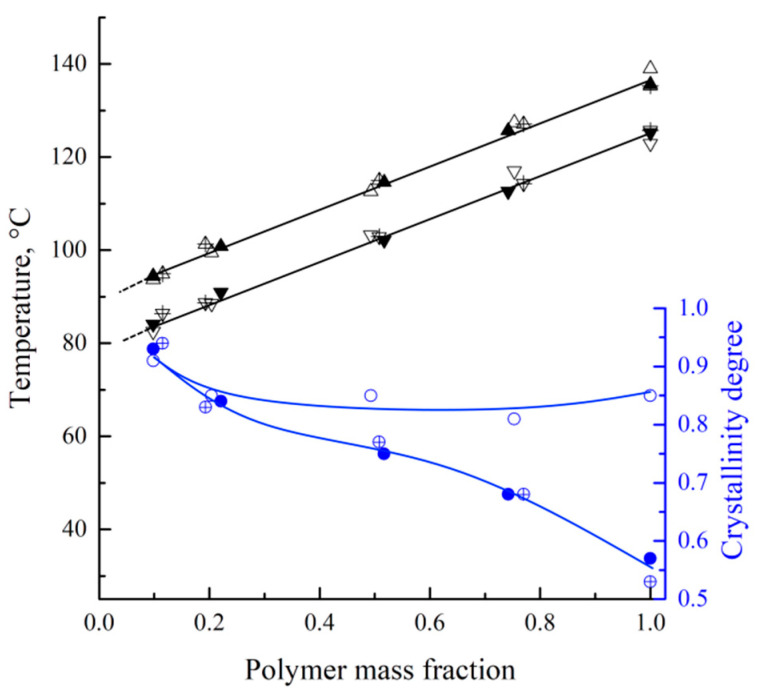
Dependencies of melting (black triangles) and crystallization (inverted black triangles) temperature and crystallinity degree (blue circles) of the LMW (hollow points), MMW (crossed points), and HMW (solid points) UHMWPE.

In Figure 4, the dependencies of the endothermic peak maximum and onset of exothermic peaks (black points, left vertical axis), together with polymer crystallinity degrees attained during the mixture’s cooling (blue points, right vertical axis), are plotted against the polymer mass fraction in the mixture. The crystallinity degrees were calculated using a modified Equation (2):

(8) $$\alpha =\;{∆H}_{cr}/\left(w_{2}{\times ∆H}_{m}^{100\%}\right)$$

where Δ*H_cr_* is an area of the exothermic peak on the DSC thermogram and *w*_2_ is the polymer mass fraction in the mixture.

The dependence of both the melting and crystallization temperatures on the mixture composition represents almost parallel straight lines. The crystallization temperature is 11 °C lower than the melting temperature, which is due to the kinetic sluggishness of the crystallization process. The absence of a horizontal section on these lines clearly indicates that decalin is a good solvent for UHMWPE. Consequently, the phase diagram of the mixture does not contain a liquid–liquid equilibrium binodal. The melting and crystallization temperatures are independent of the polymer MW, which is to be expected for the high-MW polymer samples synthesized in similar conditions.

Interestingly, an increase in the decalin mass fraction in the mixtures containing MMW and HMW leads to a gradual increase in α (from ~55 to ~92%). This is because an increase in the decalin concentration in the mixture promotes higher mobility of the macromolecules and thus enables the formation of a more ideal crystalline structure. At the same time, the α values of the LMW sample show almost no dependence on the mixture composition, being equal to ~85% for the pure polymer and slightly increasing (to ~92%) for the mixture containing ~10% wt. of the polymer. It can be assumed that the shorter macromolecules in this sample are less entangled, which enables them to reach higher values of α during cooling at a rate of 10 °C min^−1^, even in the absence of plasticizer/diluent.

The dependence of the polymer melting temperature on the mixture (Figure 4) is one of the lines presented on the phase diagram of the binary mixture. In addition, as it was shown previously for various mixtures [57], the phase diagram of the semicrystalline (SC) polymer mixture with a good solvent should contain at least one more boundary curve, reflecting a dependence of the SC polymer swelling degree on temperature (temperature dependence of low molecular liquid solubility in amorphous regions of the SC polymer).

**Figure 5:**
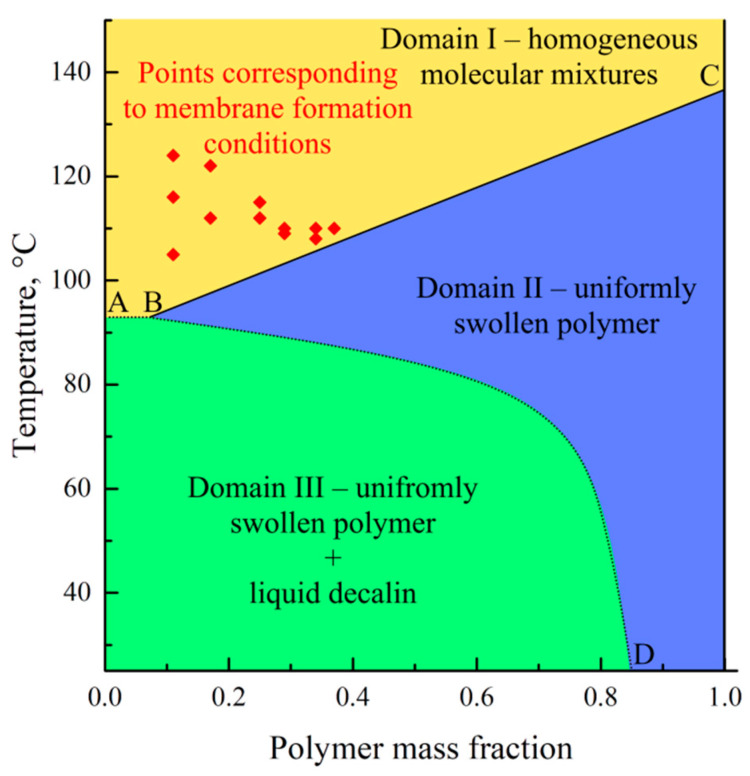
Phase diagram for the UHMWPE mixture with decalin. Red diamonds correspond to the temperature and PMFSF of the samples before termination of the swelling process by cooling.

Figure 5 shows the phase diagram containing this curve. In this diagram, the ABC curve reflects a dependence of the polymer melting temperature on the mixture composition, whereas the BD curve corresponds to the temperature dependence of the UHMWPE swelling degree in decalin. The BC line on this diagram was plotted using the DSC data obtained in the present work, while the BD curve was plotted using a dependence of the UHMWPE swelling degree on temperature reported by Zhao et al. [58]. The homogeneous molecular mixtures of the components exist in domain I. In domain II, the systems represent the uniformly swollen SC polymer (gel), while in domain III, such a gel coexists with the almost pure solvent.

The points corresponding to the temperatures of the swelling process of the monolithic films and the solvent concentration in these films at the moment of swelling termination by cooling (*w*_1_ = 1 − PMFSF) are also plotted within the temperature–composition field of the diagram. All these points are located in domain I. This means that termination of swelling the films was actually solutions of decalin in fully amorphous UHMWPE. Nevertheless, the films maintained their shape and integrity due to the very high viscosity of such solutions. Obviously, such “limited” swelling would be unlimited in time, i.e., a homogeneous solution of UHMWPE in the full volume of a decalin bath would be formed with a composition corresponding to the bath modulus (in our case 0.5% wt.) if the swelling process was not ceased by cooling.

An analysis of the phase diagrams shown in Figure 5 reveals that the cooling of the prepared swollen films leads, at first, to their entering domain II, in which the polymer starts to crystallize across the volume of the swollen film, and then to domain III, where, from the swollen SC UHMWPE, almost pure decalin segregates. The details of the structure formation mechanism were discussed in our previous work [38].

### 3.2. Morphology and Properties of the UHMWPE Monolithic Films

Prior to discussing the effect of the variation in the process parameters of the membrane formation on their structure and properties, it is useful to consider first how polymer MW affects the characteristics of the hot-pressed monolithic films used for membrane preparation.

**Figure 6:**
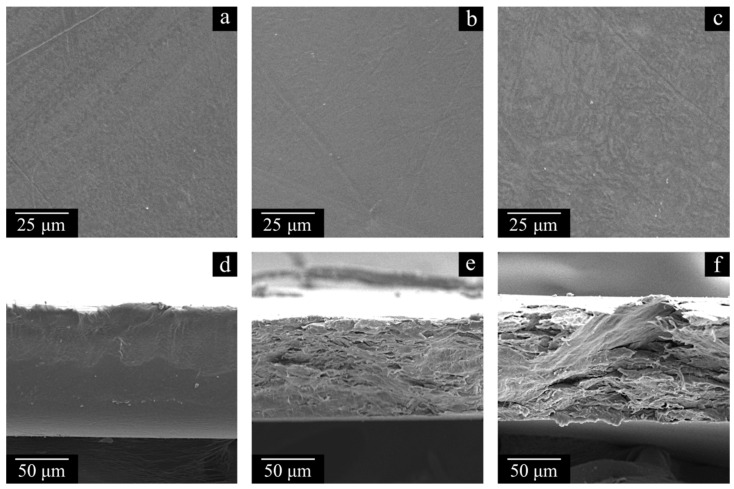
SEM images of the surface (**a**–**c**) and cross-section surface (**d**–**f**) of the monolithic films prepared from LMW (**a**,**d**), MMW (**b**,**e**), and HMW (**c**,**f**).

Figure 6 shows the SEM images of the surfaces and cross-sections of the monolithic films prepared from LMW, MMW, and HMW. The surfaces of all the films are smooth and homogeneous, which is indicative of their successful preparation. The cross-section surface of the LMW sample is significantly different from those of the MMW and HMW samples. This is due to the inability to obtain a high-quality cross-section of the sample by cleavage due to highly elastic deformation before its break, even at −196 °C, leading to the formation of fibrils and pores. Because of this, the image actually shows a cut surface of the sample instead of a cleavage surface. The MMW and HMW samples have a layered structure (Figure 6). In our opinion, this is because, for these polymers of very high MW, even at a pressure of 160 bar and a temperature of 170 °C, it was not possible to form a homogeneous polymer melt across the thickness of the samples. Meanwhile, the density measurements with a pycnometer showed that all the prepared samples were monolithic and had a density of 0.93–0.94 g cm^−3^.

Table 3 shows the results of the physico-mechanical tests of the monolithic films. It can be seen that both the σ and ε of the samples decrease with increasing MW, which is in agreement with the results of the SEM study of the films. Indeed, the samples for which we were not able to achieve complete mixing of macromolecules (formation of a homogeneous melt) are characterized by a lower σ and by a more fragile character of the break. The trends of the elasticity modulus change correlate well with the data on the crystallinity degree of the samples (Figure 4). One can see that the LMW sample, with an α value of ~85%, has a higher elasticity modulus than the MMW and HMW samples, with an α value of ca. 55%.

It is interesting to note that the films prepared from LMW also have the best abrasion resistance. Taking into account the data shown in Figure 4 and Figure 6, this can be explained by a combined effect of two factors. Firstly, this sample has a much higher crystallinity degree, determining a high abrasion resistance. Secondly, its more uniform (monolithic) structure is also favorable for high wear resistance. At the same time, the MMW sample is expectedly characterized by slightly higher mass loss after the abrasion test under the same conditions compared to the HMW sample with a similar crystallinity degree and morphology.

### 3.3. Morphology and Properties of the Prepared Membranes

It should be noted that we attempted to prepare the membrane samples from the mixtures of decalin with all three types of the UHMWPE samples chosen for study at different PMFSF values. However, it was found that during swelling, the LMW films lost 10 to 30% (depending on the swelling temperature and time) of the initial mass, while for the MMW and HMW films, the mass loss did not exceed ~2%. The samples prepared from LMW often attained a shape significantly different from the initial square, and, in some cases, lost their integrity in either the swelling, extraction, or drying stage. The reason for such a difference in the behavior of the films during swelling is obviously their different MWs.

Indeed, annealing the film in the decalin medium (at temperatures corresponding to the domain I in the phase diagram in Figure 5) makes two processes thermodynamically possible: diffusion of decalin molecules into the film and mass transfer of polymer into the decalin volume. Obviously, the latter process is more pronounced for the films composed of the LMW polymer.

Thus, we were able to prepare only one sample of a membrane suitable for the following studies of morphology, transport, and physico-mechanical properties from the LMW film (at PMFSF = 0.11) and several samples from MMW and HMW with different PMFSF values.

#### 3.3.1. Effect of Polymer MW on Structure and Properties of Membranes at Fixed PMFSF

**Figure 7:**
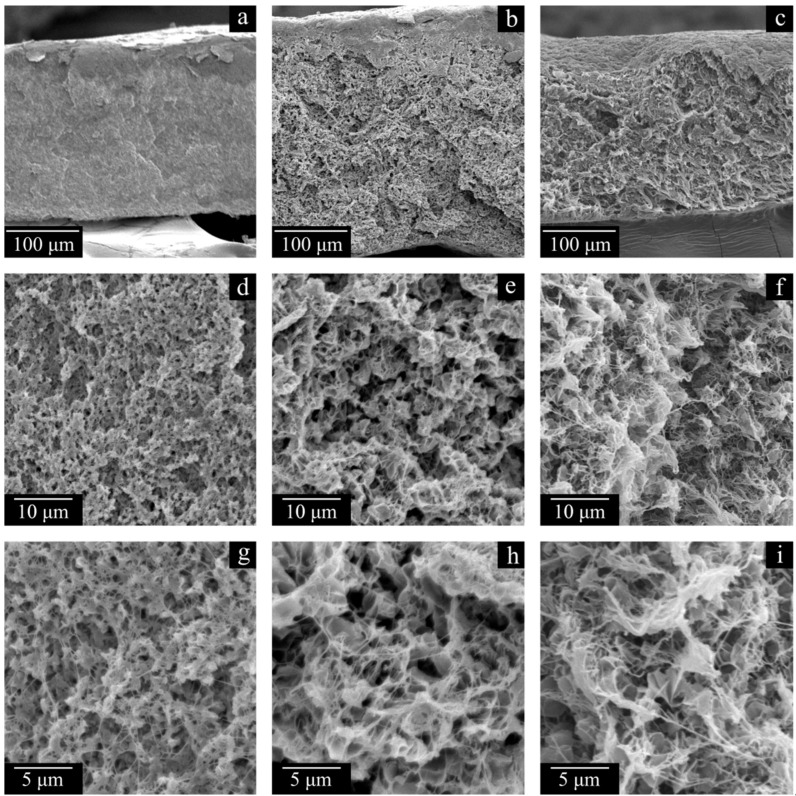
SEM images of the cross-section surface of the LMW-11 (**a**,**d**,**g**), MMW-11 (**b**,**e**,**h**), and HMW-11 (**c**,**f**,**i**) membranes.

**Figure 8:**
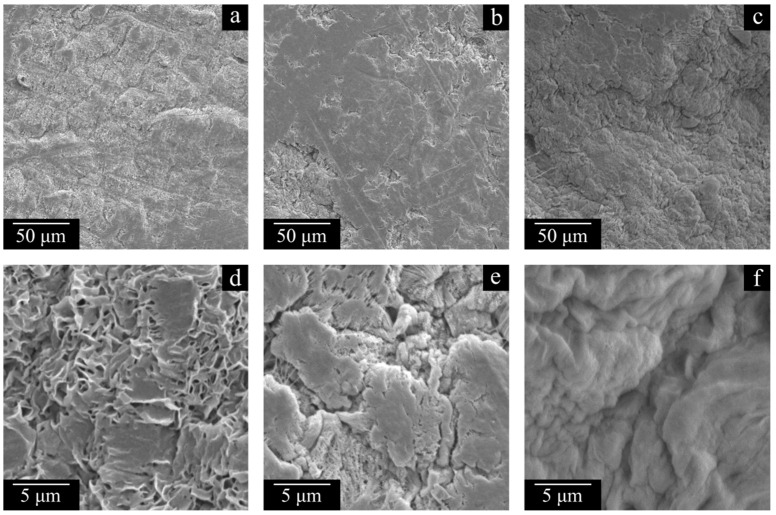
SEM images of surfaces of the LMW-11 (**a**,**d**), MMW-11 (**b**,**e**), and HMW-11 (**c**,**f**) membranes.

**Figure 9:**
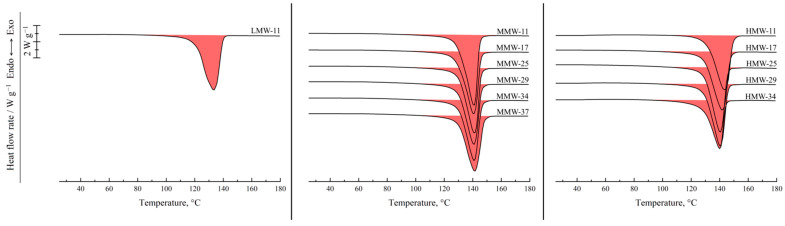
DSC-thermograms of the prepared membranes. Sample codes are shown above the curves.

**Figure 10:**
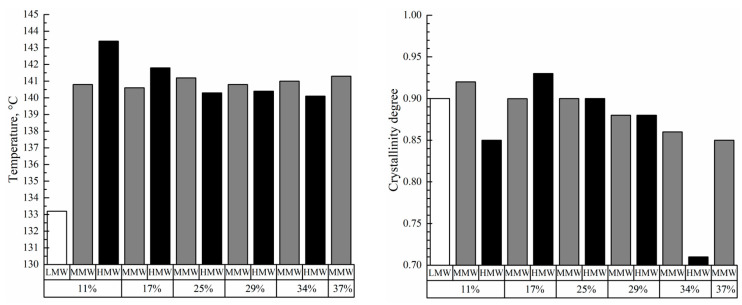
Values of the melting temperatures (**left**) and crystallinity degrees (**right**) of the prepared membranes. The polymer used for the preparation of the sample and PMFSF is indicated under the histograms.

In order to assess the effect of the polymer MW on the structure and properties of the membranes, the samples prepared with the same PMFSF of 0.11 were compared. Analysis of the data presented in Figure 7, Figure 8, Figure 9 and Figure 10 and Table 4 regarding the membrane samples prepared at PMFSF = 0.11 allows us to draw the following conclusions.

Traces of the initial powder particles are visible in the morphology of the cross-section surface of the MMW and HMW monolithic films, but they are absent in the structure of the membranes. Furthermore, the membrane structure itself is uniform across the thickness of the sample. Thus, a treatment of the films with decalin at elevated temperature ensures macromolecule mixing even in the samples where it was not complete at the stage of hot-pressing the UHMWPE powder. Further evidence of this phenomenon is the results of the studies of the membranes’ physico-mechanical properties. While the σ values of monolithic films decrease by a factor of ca. 1.5 in an LMW > MMW > HMW series and their ε values decrease in the same series by almost two orders of magnitude, the trends are opposite for the membranes prepared from these polymers. Moreover, the ε of the MMW-11 and HMW-11 samples is even higher than that of the corresponding monolithic films.

The membrane structure in all cases (Figure 7 and Figure 8), expectedly, is a result of polymer crystallization directly from its uniform mixture with decalin. The morphology of the LMW-11 sample consists of globule-like structures interconnected by thin fibrils. Taking into account the data contained in Table 4, it can be concluded that such a structure is characterized by poor mechanical properties. In the morphology of the MMW-11 and HMW-11 samples, the leaf-like formations dominate, similar to those found in other UHMWPE membranes [59]. The morphology of the cross-section of these samples also contains the fibrils that were absent in the initial sample but were formed during its cleavage in liquid nitrogen [60]. When the MMW-11 and HMW-11 samples are compared, the porosity and pore size of the latter sample are significantly lower. It should be noted that since the PMFSF values during these sample preparations were identical, their different porosity must be a result of different shrinkage at the stages of decalin extraction and drying from iso-propanol. Both the transformation of the structure from globule-like to leaf-like and the increase in the proneness of the formed porous structure to shrinkage are consequences of a polymer MW increase. On the one hand, UHMWPEs with MWs higher than 3·10^6^ g mol^−1^ are more prone to the formation of leaf-like structures during crystallization. On the other hand, a higher MW leads to increased shrinkage [61].

The structure of the sample surface is also significantly different. When the LMW-11 (Figure 8a,d) and MMW-11 (Figure 8b,e) samples are compared, the quantity and size of open pores of the latter sample are lower. Furthermore, open pores were not even observed on the surface of the HMW-11 sample due to the insufficient resolving power of the SEM device. It can also be noted that the surface of this sample is wrinkled, probably due to nonuniform shrinkage.

The tendencies of the changes in the transport properties (mean pore size and permeance) are in good agreement with the above-described trends of changes in overall porosity and surface porosity. The LMW-11 sample demonstrates a higher pore size and permeance than the MMW-11 sample, while the latter sample exhibits the smallest pore size and permeance (Table 4). For the samples prepared from the same polymer, the pore size follows porosity, and hence, more porous samples possessed higher MFP and permeance. The rejection of blue dextran also well correlates with MFP. For the dense HMW-11 sample, the rejection was the highest (up to 98%) when the more porous sample LMW-11 possessed a lower rejection of 73%.

Figure 9 shows the DSC thermograms of the membrane samples used to assess the melting temperature of the polymer in these membranes and their crystallinity degree. All the thermograms are characterized by a single endothermic peak reflecting amorphization of UHMWPE. The melting temperature of the membrane samples increases with the increase in polymer MW (Figure 10). Furthermore, if these values are compared with those of UHMWPE in the monolithic films (Figure 2 and Figure 4), it can be concluded that the melting temperature decreases for the LMW sample by ~6 °C, while it increases by ~6 and ~8 °C for the MMW and HMW samples, respectively. The crystallinity degree of UHMWPE in the membranes as a function of polymer MW passes through a quite pronounced maximum (Figure 10). At the same time, the crystallinity degree of UHMWPE in its mixtures with decalin, containing 10% wt. of the polymer (Figure 2, Figure 3 and Figure 4), is almost the same for all three studied samples. However, it should be noted that the mixtures in the DSC experiment were cooled from 180 °C, while in the membrane preparation, they were cooled from 105 to 124 °C depending on the sample (Table 2). Bearing in mind the data on the morphology of the samples, it can be concluded that in the LMW-11 samples, an entirely new crystalline structure was formed with no traces of the initial crystalline structure of the monolithic film. On the one hand, the presence of decalin creates more favorable conditions for reaching a higher crystallinity degree than that in the initial film. On the other hand, in these conditions, more stressed and defective crystallites were formed that melt at lower temperatures. An increase in the melting temperature with the increase in polymer MW indicates that UHMWPE with a higher MW is able to form a more ideal crystalline structure. It can be assumed that the lower crystallinity degree of the HMW-11 sample is a result of high shrinkage that, probably, led to lamellae fragmentation, as it was shown in ref. [62].

The polymer MW increase leads to an increase in the σ of the membrane samples prepared at PMFSF = 0.11. At the same time, their ε passes through a maximum. The LMW-11 sample has very low mechanical properties, which is attributed to the peculiarities of its structure that significantly differ from the structure of the other samples (see Figure 7a,d,g). The MMW-11 sample has lower tensile strength but higher elongation at break than the HMW-11 sample. Furthermore, the ε of the porous samples is about an order of magnitude higher than that of the corresponding monolithic films. This is due to the fact that the treatment of the films in decalin favors effective mixing of macromolecules originating from different particles of the polymer powder across the film volume and thus the formation of a continuous, uniform matrix. A higher σ value of the MMW-11 sample compared to the HMW-11 sample is a result of a porosity decrease.

The trends observed in the wear resistance studies of the prepared samples are in good agreement with the conclusions discussed above. Particularly, the LMW-11 sample with low mechanical properties quickly collapsed in the abrasion experiments, while the MMW-11 sample and the HMW-11 sample demonstrate excellent wear resistance that is even better than that of the corresponding monolithic films (Table 3). This is obviously due to an increase in the crystallinity degree upon transformation of monolithic films into porous membrane samples. Despite the differences in porosity of the MMW-11 sample and the HMW-11 sample, their wear resistance is almost the same within the experimental error. Apparently, the combined effect of increased MW and lower porosity, which should lead to better abrasion resistance, is compensated for by its deterioration due to lower crystallinity degree.

#### 3.3.2. Effect of PMFSF and Polymer MW on the Structure and Properties of the Membranes

**Figure 11:**
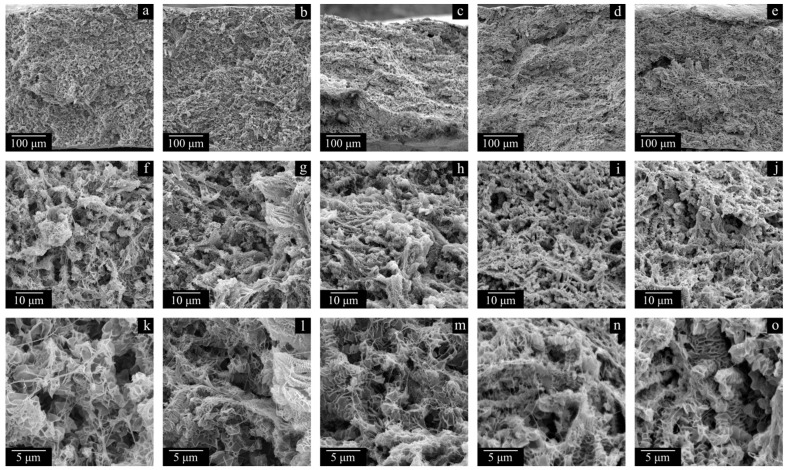
SEM images of the cross-section surfaces of the membranes MMW-17 (**a**,**f**,**k**), MMW-25 (**b**,**g**,**l**), MMW-29 (**c**,**h**,**m**), MMW-34 (**d**,**i**,**n**), and MMW-37 (**e**,**j**,**o**).

**Figure 12:**
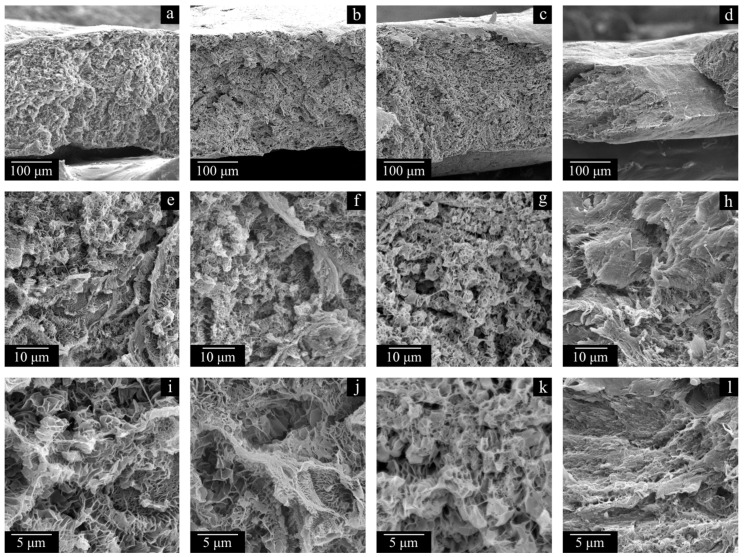
SEM images of the cross-section surface of the membranes HMW-17 (**a**,**e**,**i**), HMW-25 (**b**,**f**,**j**), HMW-29 (**c**,**g**,**k**), and HMW-34 (**d**,**h**,**l**).

Figure 11 and Figure 12 show the SEM images of the cross-section surfaces of the membranes prepared from decalin mixtures with MMW and HMW at PMFSF higher than 0.11. Analysis of these images and the DSC data shown in Figure 9 and Figure 10 and Table 4 allows drawing a conclusion.

The morphology of the cross-section surfaces of all the samples except HMW-34 is similar: they are composed of leaf-like and shish-kebab structures. The experimentally found differences in porosity (Table 4) are almost indiscernible in the SEM images of the cross-section surfaces since even at −196 °C, ideally brittle cleavage of the samples is not possible. A rather unpronounced dependence of porosity on PMFSF is explained by an increase in the sample shrinkage with an increase in PMFSF in the studied range. Only the HMW-34 sample has significantly lower porosity. This means that the effect of lower shrinkage could not compensate for a decrease in porosity due to an increase in PMFSF. In the structure of this sample, both leaf-like and shish-kebab structures are almost absent due to its lower porosity compared to the other samples.

A PMFSF increase in the discussed range does not lead to significant changes in the melting temperature of both samples prepared from MMW and HMW. At the same time, the crystallinity degree decreases as PMFSF increases. In our opinion, this is because a decalin content increase in the swollen films makes macromolecules more mobile and thus makes it possible to achieve a higher crystallinity degree. The significantly lower crystallinity degree of the HMW-34 sample is probably explained by the fact that the swelling conditions were too mild to fully erase the memory of the initial crystalline structure in the sample. Thus, upon its cooling, a network of crystallites, similar to that in the monolithic film, was formed. This assumption also agrees with the abovementioned differences in the morphology of the samples (cf. Figure 12d,h,l and, for example, Figure 12c,g,k).

The transport properties of the obtained membranes depend on two factors. The first factor is polymer MW. The membranes obtained with the same PMFSF demonstrate a tendency to increase MFP and permeance with decreasing MW (Table 4). The second factor is membrane porosity. More porous samples possess a higher pore size and permeance. The highest permeance of 107 L m^−2^ h^−1^ bar^−1^ was observed for the most porous MMW-29 sample. This sample also possesses a higher pore size MFP of 50 nm. The HMW-34 and HMW-11 samples with a low porosity demonstrate a low pore size of 15 and 25 nm, respectively. The permeance of these samples is expectedly low at 1.4 and 17 L m^−2^ h^−1^ bar^−1^. Consequently, rejection of the blue dextran dye completely followed expectations based on their pore size. The highest rejection of 98% was observed for the densest HMW-11 sample, while the most porous MMW-29 sample with the highest pore size possessed the lowest rejection of 72%.

In the discussed range of PMFSF, the σ value of the samples prepared from MMW monotonically increases with an increase in PMFSF, while that of the samples prepared from HMW passes through a minimum. Such a character of the tensile strength variation is due to their different porosities and crystallinity degrees. An increase in the crystallinity degree usually leads to higher σ values. In this case, when the crystallinity degree is ca. 90% and higher, its increase probably decreases the number of tie chains that transfer the external stress between the crystallites and thus makes the sample less robust. This is why, in our opinion, in the series of the MMW-17, MMW-25, MMW-29, MMW-34, and MMW-37 samples with almost the same porosity but decreasing crystallinity degree, the tensile strength increases. The same can be said about the HMW-17, HMW-25, and HMW-29 samples. The higher σ values of the HMW-11 and HMW-34 samples compared to those of the HMW-17, HMW-25, and HMW-29 samples are explained by the lower porosity of the former. In turn, the low porosity of the HMW-11 sample is a result of its very high shrinkage, whereas, for the HMW-34 sample, it is a consequence of its initially high PMFSF.

The wear resistance of the samples also correlates well with the data on the crystallinity degree of the samples. Particularly, almost all the discussed samples have the same (within experimental error) values of mass loss. The only exception is the HMW-34 sample with a significantly lower crystallinity degree. It should once again be noted that the wear resistance of the samples was assessed using a modified method (five times higher than the standard pressing load and four times the distance (200 cycles of sample moving)) since the standard method [26,52] yielded almost negligible values of mass loss. This means that the prepared samples have a great wear resistance, much higher than that reported in the literature for the membrane samples prepared from various polymers, since UHMWPE has a substantial advantage over other polymers in this regard.

Before concluding, it is interesting to discuss why the porosity and some transport properties of the samples with an increase in PMFSF pass through a maximum and their tensile strength and rejection pass through a minimum (Table 4). Obviously, the variation in porosity, mean pore size, and permeance is a result of competition between two factors: the quantity of decalin in the swollen film (*w*_1_ = 1 − PMFSF), which increases porosity, and shrinkage, which results in a decrease in porosity. Our experiments show that shrinkage increases with an increase in *w*_1_. The observed trend in the σ variation is also a result of competition between these two factors, and it is also affected by the crystallinity degree.

Indeed, at low PMFSF, despite the high amount of decalin in the swollen film, high shrinkage leads to a significant decrease in overall porosity and thus to high values of tensile strength. An increase in PMFSF makes the shrinkage effect on porosity less pronounced, so that the samples, even at a lower decalin content in the swollen film, are more porous and, consequently, less strong. Although a subsequent increase in the PMFSF value continues to diminish the shrinkage effect on membrane porosity, the latter decreases because of the increase in the decalin content in the swollen film.

This observation can also explain why the membranes possess a uniform pore structure across their thicknesses (see Figure 7a–c, Figure 11a–e and Figure 12a–d). The swelling, as expected, is probably nonuniform, and the polymer concentration actually is the lowest near the surface of the film and the highest in the middle of the film. However, after the swelling process is stopped, in the stage of decalin extraction and drying the membranes of the extractant, the outer (more swollen) layers of the membrane shrink more while the inner (less swollen) layers shrink less. This ultimately leads to uniform porosity across the film thickness.

A decrease in the crystallinity degree of the membranes with the increase in PMFSF probably increases the amount of tie chains transferring the external load and thus increases tensile strength. However, according to the obtained dependencies, σ = f(PMFSF) and P = f(PMFSF), this factor is not a decisive one. It should also be noted that the proneness of the samples to shrinkage was found to increase with an increase in polymer MW. This is due to the higher stresses of the longer macromolecules that participate in a higher number of entanglements and their relaxation after removal of non-compressible liquid (solvent or extractant) from the pores.

## 4. Conclusions

In this work, the polymer MW effect on the structure and properties of membranes prepared via the controlled swelling of the monolithic UHMWPE film method was assessed for the first time. It was shown that the method allows for preparing membranes with a uniform porous structure, even if the monolithic films prepared via hot pressing have defects due to the very high viscosity of the melt. It was established that the formation of high-quality membranes by this method is impossible at a polymer MW of 0.7·10^6^ g mol^−1^ or lower. The abrasion resistance of the UHMWPE membranes was also assessed for the first time. It was shown that all the samples had excellent tribological properties, which supports their recommendation for the filtration of highly abrasive suspensions. It was also found that the abrasion resistance was almost independent of the MW of the polymer and membrane porosity for the studied samples, but it increased with an increase in the crystallinity degree. The effect of the polymer concentration in the swollen films on the structure and properties of the membranes was studied. It was established that with an increase in PMFSF, the tensile strength of the membranes passes through a minimum, whereas porosity, mean pore size, and permeance pass through a maximum, while the shrinkage and crystallinity degree decrease. The explanation of such dependencies was proposed. Analysis of the SEM images of the prepared samples showed that all the samples have a morphology consisting of leaf-like formations and shish-kebab structures.

## Figures and Tables

**Table 1:** Principal characteristics of the synthesized UHMWPE samples and hot-pressed monolithic films.

Sample	Weight Average MW, g mol^−1^	Melting Temperature *	Crystallinity Degree (α) *, %
LMW	700,000	139.0	85.6
MMW	3,700,000	135.3	53.7
HMW	4,700,000	135.6	55.3

* Melting temperature and crystallinity degree was measured for the prepared monolithic films.

**Table 2:** The membrane sample preparation conditions.

Sample Code	Polymer MW, 10^6^ g mol^−1^	Polymer Mass raction in Swollen Film (PMFSF), g g^−1^	Swelling Temperature, °C	Swelling Time, min
LMW-11	0.7	0.11	105	20
MMW-11	3.7	0.11	124	100
MMW-17	3.7	0.17	122	40
MMW-25	3.7	0.25	115	90
MMW-29	3.7	0.29	110	60
MMW-34	3.7	0.34	110	55
MMW-37	3.7	0.37	110	50
HMW-11	4.7	0.11	116	80
HMW-17	4.7	0.17	112	30
HMW-25	4.7	0.25	112	25
HMW-29	4.7	0.29	109	30
HMW-34	4.7	0.34	108	20

**Table 3:** Physico-mechanical properties of the monolithic films.

Sample Code	σ, MPA	ε, %	Elasticity Modulus, MPa	Mass Loss After Abrasion Test, g m^−2^
LMW	37 ± 0.8	700 ± 40	780 ± 50	12 ± 2
MMW	34.1 ± 0.8	22 ± 2	520 ± 30	20 ± 2
HMW	23.3 ± 0.4	7.5 ± 0.8	570 ± 20	18 ± 2

**Table 4:** Physico-mechanical, transport, and other characteristics of the membranes.

Sample Code	σ, MPA	ε, %	Porosity, %	MFP, nm	Permeance, L m^−2^ h^−1^ bar^−1^	*R*, %	Mass Loss After Abrasion Test, g m^−2^
LMW-11	1.0 ± 0.2	9 ± 0.6	60 ± 4	47 ± 5	75 ± 3	73 ± 3	42 ± 5
MMW-11	6.3 ± 0.8	190 ± 25	65 ± 4	36 ± 4	58 ± 3	92.7 ± 1.5	10 ± 1
MMW-17	7.2 ± 0.7	170 ± 20	67 ± 4	39 ± 3	50 ± 2	86 ± 2	11 ± 1
MMW-24	10.1 ± 0.7	180 ± 30	72 ± 5	43 ± 6	66 ± 4	77 ± 3	10 ± 1
MMW-29	11.0 ± 0.8	160 ± 35	74 ± 6	50 ± 4	107 ± 6	72 ± 4	11 ± 1
MMW-34	13.8 ± 1.2	170 ± 30	69 ± 5	41 ± 4	59 ± 5	82 ± 3	11 ± 1
MMW-37	17.8 ± 1.1	130 ± 20	67 ± 7	38 ± 5	47 ± 5	90.4 ± 1.8	11 ± 1
HMW-11	11.9 ± 0.9	125 ± 20	29 ± 3	15 ± 2	1.4 ± 0.2	98.2 ± 0.8	10 ± 1
HMW-17	5.0 ± 0.6	100 ± 15	72 ± 4	39 ± 4	54 ± 3	83 ± 3	11 ± 1
HMW-24	5.6 ± 0.8	90 ± 15	71 ± 4	37 ± 5	50 ± 4	90.7 ± 1.6	11 ± 1
HMW-29	10.6 ± 1.0	105 ± 25	67 ± 4	34 ± 3	44 ± 3	94.7 ± 1.4	10 ± 1
HMW-34	13.6 ± 1.2	45 ± 6	49 ± 5	25 ± 3	17 ± 2	96.9 ± 1.1	14 ± 1

## Data Availability

The raw data supporting the conclusions of this article will be made available by the authors on request.

## References

[1] Kang G.D., Cao Y.M. (2014). Application and Modification of Poly(Vinylidene Fluoride) (PVDF) Membranes—A Review. J. Membr. Sci..

[2] Volkov A., Yushkin A., Kachula Y., Khotimsky V., Volkov V. (2014). Application of Negative Retention in Organic Solvent Nanofiltration for Solutes Fractionation. Sep. Purif. Technol..

[3] Ezugbe E.O., Rathilal S. (2020). Membrane Technologies in Wastewater Treatment: A Review. Membranes.

[4] Wen Y., Yuan J., Ma X., Wang S., Liu Y. (2019). Polymeric Nanocomposite Membranes for Water Treatment: A Review. Environ. Chem. Lett..

[5] Li L., Duan Y. (2023). Engineering Polymer-Based Porous Membrane for Sustainable Lithium-Ion Battery Separators. Polymers.

[6] Jiang X., Shao Y., Sheng L., Li P., He G. (2021). Membrane Crystallization for Process Intensification and Control: A Review. Engineering.

[7] Vladisavljević G.T. (2024). Preparation of Microparticles and Nanoparticles Using Membrane-Assisted Dispersion, Micromixing, and Evaporation Processes. Particuology.

[8] Pochivalov K.V., Basko A.V., Lebedeva T.N., Yurov M.Y., Yushkin A.A., Bronnikov S.V., Volkov A.V. (2024). PVDF Membrane Formation via NIPS in Isothermal and Non-Isothermal Conditions: Thermodynamics, Structure, and Properties. Membr. Membr. Technol..

[9] Serbanescu O.S., Voicu S.I., Thakur V.K. (2020). Polysulfone Functionalized Membranes: Properties and Challenges. Mater. Today Chem..

[10] Wang H.H., Jung J.T., Kim J.F., Kim S., Drioli E., Lee Y.M. (2019). A Novel Green Solvent Alternative for Polymeric Membrane Preparation via Nonsolvent-Induced Phase Separation (NIPS). J. Membr. Sci..

[11] Kammakakam I., Lai Z. (2023). Next-Generation Ultrafiltration Membranes: A Review of Material Design, Properties, Recent Progress, and Challenges. Chemosphere.

[12] Yang S., Xiao C., Huang Y., Ji D., Chen K. (2022). Effect of Additive and Coagulation Bath Temperature on Structure and Properties of HDPE Membranes via Thermally Induced Phase Separation. J. Mater. Sci..

[13] Pochivalov K., Basko A., Yurov M., Lebedeva T., Shalygin M., Lavrentyev V., Yushkin A., Anokhina T., Volkov A. (2024). Polypropylene Membranes Prepared via Non-Solvent/Thermally Induced Phase Separation: Effect of Non-Solvent Nature. J. Membr. Sci..

[14] Tang Y., Li M., Lin Y., Wang L., Wu F., Wang X. (2021). A Novel Green Diluent for the Preparation of Poly(4-Methyl-1-Pentene) Membranes via a Thermally-Induced Phase Separation Method. Membranes.

[15] Basko A., Pochivalov K. (2022). Current State-of-the-Art in Membrane Formation from Ultra-High Molecular Weight Polyethylene. Membranes.

[16] Ding L., Li D., Du F., Zhang D., Zhang S., Wu T. (2023). Novel Preparation of Lithium-ion Battery Wet-processed Separator Based on the Synergistic Effect of Porous Skeleton Nano-Al_2_O_3_ in Situ Blending and Synchro-draw. Polym. Int..

[17] Dayyoub T., Maksimkin A., Olifirov L.K., Chukov D., Kolesnikov E., Kaloshkin S.D., Telyshev D.V. (2023). Structural, Mechanical, and Tribological Properties of Oriented Ultra-High Molecular Weight Polyethylene/Graphene Nanoplates/Polyaniline Films. Polymers.

[18] Cicek N., Dionysiou D., Suidan M.T., Ginestet P., Audic J.M. (1999). Performance Deterioration and Structural Changes of a Ceramic Membrane Bioreactor Due to Inorganic Abrasion. J. Membr. Sci..

[19] Mozia S., Szymański K., Michalkiewicz B., Tryba B., Toyoda M., Morawski A.W. (2015). Effect of Process Parameters on Fouling and Stability of MF/UF TiO2 Membranes in a Photocatalytic Membrane Reactor. Sep. Purif. Technol..

[20] Chesters S.P., Pena N., Gallego S., Fazel M., Armstrong M.W., del Vigo F. (2013). Results from 99 Seawater RO Membrane Autopsies. IDA J. Desalin. Water Reuse.

[21] Chen S., Zhou J., Li K., Chen Z., Li X., Su X., Ao X., Xie H., Chen L., Wu X. (2023). Superhydrophobic and Robust Photothermal/Electrothermal PVDF-a/CNT-S@PDMS Membrane for Crude Oil Removal and Dye Adsorption. Compos. Sci. Technol..

[22] Lai C.Y., Groth A., Gray S., Duke M. (2014). Preparation and Characterization of Poly(Vinylidene Fluoride)/Nanoclay Nanocomposite Flat Sheet Membranes for Abrasion Resistance. Water Res..

[23] Lai C.Y., Groth A., Gray S., Duke M. (2013). Enhanced Abrasion Resistant PVDF/Nanoclay Hollow Fibre Composite Membranes for Water Treatment. J. Membr. Sci..

[24] Ji J., Zhou S., Lai C.Y., Wang B., Li K. (2015). PVDF/Palygorskite Composite Ultrafiltration Membranes with Enhanced Abrasion Resistance and Flux. J. Membr. Sci..

[25] Arimi M.M., Namango S.S., Götz G., Zhang Y., Kiriamiti K., Geißen S.U. (2016). The Abrasion Effects of Natural Organic Particles on Membrane Permeability and the Size Distribution of Recalcitrants in a Colored Effluent. J. Membr. Sci..

[26] Liao Y., Zheng G., Huang J.J., Tian M., Wang R. (2020). Development of Robust and Superhydrophobic Membranes to Mitigate Membrane Scaling and Fouling in Membrane Distillation. J. Membr. Sci..

[27] Khakpour S., Jafarzadeh Y., Yegani R. (2019). Incorporation of Graphene Oxide/Nanodiamond Nanocomposite into PVC Ultrafiltration Membranes. Chem. Eng. Res. Des..

[28] Behboudi A., Jafarzadeh Y., Yegani R. (2016). Preparation and Characterization of TiO_2_ Embedded PVC Ultrafiltration Membranes. Chem. Eng. Res. Des..

[29] Behboudi A., Jafarzadeh Y., Yegani R. (2017). Polyvinyl Chloride/Polycarbonate Blend Ultrafiltration Membranes for Water Treatment. J. Membr. Sci..

[30] Wang N., Raza A., Si Y., Yu J., Sun G., Ding B. (2013). Tortuously Structured Polyvinyl Chloride/Polyurethane Fibrous Membranes for High-Efficiency Fine Particulate Filtration. J. Colloid Interface Sci..

[31] Xiao Q.R., Sun S. (2023). An Anti-Abrasion Sandwich Structured Membrane for Highly Efficient Oil/Water Separation. Polym. Adv. Technol..

[32] Ye X., Huang R., Zheng Z., Liu J., Chen J., Lv Y. (2024). Anti-Abrasion Collagen Fiber-Based Membrane Functionalized by UiO-66-NH2 with Ultra-High Efficiency and Stability for Oil-in-Water Emulsions Separation. Chin. J. Chem. Eng..

[33] Han M., Liu G., Fang C., Zhu L. (2024). Robust Superhydrophobic Poly(Vinylidene Fluoride) Membranes with Spherulitic Surface Morphology against Wetting and Scaling in Membrane Distillation. J. Membr. Sci..

[34] Lin X., Hong J. (2019). Recent Advances in Robust Superwettable Membranes for Oil–Water Separation. Adv. Mater. Interfaces.

[35] Cao X., Li Y., He G. (2020). Fabrication of Self-Lubricating Porous UHMWPE with Excellent Mechanical Properties and Friction Performance via Rotary Sintering. Polymers.

[36] Zhang Q., Lan L., Zheng Z., Liu P., Wu H., Guo S., Lin C., He G. (2022). Constructing Highly Oriented and Condensed Shish-Kebab Crystalline Structure of HDPE/UHMWPE Blends via Intense Stretching Process: Achieving High Mechanical Properties and in-Plane Thermal Conductivity. Polymer.

[37] Quan J., Yu J., Wang Y., Hu Z. (2022). Construction of Intrinsic Superhydrophobic Ultra-High Molecular Weight Polyethylene Composite Membrane for DCMD. J. Membr. Sci..

[38] Pochivalov K., Basko A., Lebedeva T., Yurov M., Yushkin A., Volkov A., Bronnikov S. (2023). Controlled Swelling of Monolithic Films as a Facile Approach to the Synthesis of UHMWPE Membranes. Membranes.

[39] Matsuyama H., Maki T., Teramoto M., Asano K. (2002). Effect of Polypropylene Molecular Weight on Porous Membrane Formation by Thermally Induced Phase Separation. J. Membr. Sci..

[40] Atkinson P.M., Lloyd D.R. (2000). Anisotropic Flat Sheet Membrane Formation via TIPS: Atmospheric Convection and Polymer Molecular Weight Effects. J. Membr. Sci..

[41] Yave W., Quijada R., Serafini D., Lloyd D. (2005). Effect of the Polypropylene Type on Polymer–Diluent Phase Diagrams and Membrane Structure in Membranes Formed via the TIPS ProcessPart I. Metallocene and Ziegler–Natta Polypropylenes. J. Membr. Sci..

[42] Hassankiadeh N.T., Cui Z., Kim J.H., Shin D.W., Sanguineti A., Arcella V., Lee Y.M., Drioli E. (2014). PVDF Hollow Fiber Membranes Prepared from Green Diluent via Thermally Induced Phase Separation: Effect of PVDF Molecular Weight. J. Membr. Sci..

[43] Zhang P., Fang C., Rajabzadeh S., Liu W., Jia Y., Shen Q., Zhang L., Wang S., Kato N., Matsuyama H. (2021). Effect of Polymer Molecular Weight on Structure and Performance of PVDF Hollow Fiber Membranes Prepared via TIPS Process with Co-Extrusion of Solvent Using Triple Orifice Spinneret. J. Membr. Sci..

[44] Deplancke T., Lame O., Rousset F., Seguela R., Vigier G. (2015). Mechanisms of Chain Reentanglement during the Sintering of UHMWPE Nascent Powder: Effect of Molecular Weight. Macromolecules.

[45] Vadivel H.S., Bek M., Šebenik U., Perše L.S., Kádár R., Emami N., Kalin M. (2021). Do the Particle Size, Molecular Weight, and Processing of UHMWPE Affect Its Thermomechanical and Tribological Performance?. J. Mater. Res. Technol..

[46] Cao C., Jiang W., Lin Y., Chen X., Qian Q., Chen Q., Yu D., Chen X. (2020). Sensitive Phase Separation Behavior of Ultra-High Molecular Weight Polyethylene in Polybutene. Polym. Test..

[47] Yang Y., Sheng L., Zhang H., Li M.L., Xu R., Bai Y., Song S., Liu G., Wang T., Huang X. (2022). Investigation on the Solid–Liquid (S–L) Phase Separation of the PE/LP Blend with Different Molecular Weight Polyethylene. Polym. Bull..

[48] Liu R., Wang X., Yu J., Wang Y., Zhu J., Hu Z. (2016). Development and Evaluation of UHMWPE/Woven Fabric Composite Microfiltration Membranes via Thermally Induced Phase Separation. RSC Adv..

[49] Basko A.V., Lebedeva T.N., Yurov M.Y., Zabolotnov A.S., Gostev S.S., Gusarov S.S., Pochivalov K.V. (2024). Thermally Induced Phase Separation of UHMWPE Mixture with Dioctyl Adipate: Competition of Liquid–Liquid Phase Separation and Polymer Crystallization. Thermochim. Acta.

[50] Mirabella F.M., Bafna A. (2002). Determination of the Crystallinity of Polyethylene/α-Olefin Copolymers by Thermal Analysis: Relationship of the Heat of Fusion of 100% Polyethylene Crystal and the Density. J. Polym. Sci. Part B Polym. Phys..

[51] Yushkin A.A., Efimov M.N., Malakhov A.O., Karpacheva G.P., Bondarenko G., Marbelia L., Vankelecom I.F.J., Volkov A.V. (2021). Creation of Highly Stable Porous Polyacrylonitrile Membranes Using Infrared Heating. React. Funct. Polym..

[52] Yin Z., Yuan F., Li M., Xue M., Zhou D., Chen Y., Liu X., Luo Y., Hong Z., Xie C. (2021). Self-Cleaning, Underwater Writable, Heat-Insulated and Photocatalytic Cellulose Membrane for High-Efficient Oil/Water Separation and Removal of Hazardous Organic Pollutants. Prog. Org. Coatings.

[53] Wang Y., Fu J., Song Q., Yu J., Wang Y., Hu Z. (2022). Regulating the Dissolving System of Ultra-high Molecular Weight Polyethylene to Enhance the High-strength and High-modulus Properties of Resultant Fibers. J. Appl. Polym. Sci..

[54] Brem A., Lhost O., Tervoort T.A. (2020). Influence of Solvent Quality and Crystallization Conditions on the Drawability of Ultra-High Molecular Weight Polyethylene Cast from Solution. Macromolecules.

[55] Lemstra P.J. (2022). Chapter 1: High-Performance Polyethylene Fibers. Adv. Ind. Eng. Polym. Res..

[56] Ding H., Tian Y., Wang L., Liu B. (2007). Preparation of Ultrahigh-Molecular-Weight Polyethylene Membranes via a Thermally Induced Phase-Separation Method. J. Appl. Polym. Sci..

[57] Pochivalov K.V., Basko A.V., Kudryavtsev Y.V. (2020). Binary Mixtures of Semicrystalline Polymers with Low-Molecular-Mass Compounds: Thermal Behaviour and Phase Structure. Russ. Chem. Rev..

[58] Yu Q., Gao J., Wang Z., Feng X., Zhao Y., Chen L. (2023). Excellent Interfacial Bonding in UHMWPE Fiber/ Epoxy Resin Composites Fabricated via a Swelling Approach. J. Polym. Res..

[59] Basko A., Pochivalov K., Yurov M., Lebedeva T., Novikov I., Pakalnis V., Kuryndin I., Bronnikov S., Zabolotnov A., Yushkin A. (2025). Preparation of Porous Ultrahigh Molecular Weight Polyethylene Separators for Li-Ion Batteries via Thermally Induced Phase Separation Method. J. Ind. Eng. Chem..

[60] Sui Y., Li J., Qiu Z., Cui Y., Cong C., Meng X., Ye H., Zhou Q. (2022). Effects of the Sintering Temperature on the Superior Cryogenic Toughness of Ultra-High Molecular Weight Polyethylene (UHMWPE). Chem. Eng. J..

[61] Capaccio G., Ward I.M. (1982). Shrinkage, Shrinkage Force and the Structure of Ultra High Modulus Polyethylenes. Colloid Polym. Sci..

[62] Pakhomov P., Khizhnyak S., Reuter H., Lechner M., Tshmel A. (2003). Gel-to-Solid Transition in Polyethylene from the Viewpoint of the Crystallization Process. Macromolecules.

